# Value of Dynamic Susceptibility Contrast Perfusion MRI in the Acute Phase of Transient Global Amnesia

**DOI:** 10.1371/journal.pone.0122537

**Published:** 2015-03-24

**Authors:** Alex Förster, Mansour Al-Zghloul, Hans U. Kerl, Johannes Böhme, Bettina Mürle, Christoph Groden

**Affiliations:** Department of Neuroradiology, Universitätsmedizin Mannheim, University of Heidelberg, Mannheim, Germany; Julius-Maximilians-Universität Würzburg, GERMANY

## Abstract

**Purpose:**

Transient global amnesia (TGA) is a transitory, short-lasting neurological disorder characterized by a sudden onset of antero- and retrograde amnesia. Perfusion abnormalities in TGA have been evaluated mainly by use of positron emission tomography (PET) or single-photon emission computed tomography (SPECT). In the present study we explore the value of dynamic susceptibility contrast perfusion-weighted MRI (PWI) in TGA in the acute phase.

**Methods:**

From a MRI report database we identified TGA patients who underwent MRI including PWI in the acute phase and compared these to control subjects. Quantitative perfusion maps (cerebral blood flow (CBF) and volume (CBV)) were generated and analyzed by use of Signal Processing In NMR-Software (SPIN). CBF and CBV values in subcortical brain regions were assessed by use of VOI created in FIRST, a model-based segmentation tool in the Oxford Centre for Functional Magnetic Resonance Imaging of the Brain (FMRIB) Software Library (FSL).

**Results:**

Five TGA patients were included (2 men, 3 women). On PWI, no relevant perfusion alterations were found by visual inspection in TGA patients. Group comparisons for possible differences between TGA patients and control subjects showed significant lower rCBF values bilaterally in the hippocampus, in the left thalamus and globus pallidus as well as bilaterally in the putamen and the left caudate nucleus. Correspondingly, significant lower rCBV values were observed bilaterally in the hippocampus and the putamen as well as in the left caudate nucleus. Group comparisons for possible side differences in rCBF and rCBV values in TGA patients revealed a significant lower rCBV value in the left caudate nucleus.

**Conclusions:**

Mere visual inspection of PWI is not sufficient for the assessment of perfusion changes in TGA in the acute phase. Group comparisons with healthy control subjects might be useful to detect subtle perfusion changes on PWI in TGA patients. However, this should be confirmed in larger data sets and serial PWI examinations.

## Introduction

Transient global amnesia (TGA) is a neurological disorder characterized by a sudden onset of antero- and retrograde amnesia, and a complete recovery from this cognitive disturbance within 24 hours. The underlying pathophysiology is still unknown, but spreading depression, ischemic stroke, and venous congestion have been suggested as possible pathomechanisms. Since its first description in the 1950ies [1,2] neurologists were fascinated by the unique clinical syndrome and as a consequence, the relatively rare and benign neurological disease has attracted much interest. In the last decades, there have been many clinical studies using different diagnostic techniques in order to disclose the secret of TGA. While the hypothesis that TGA might be caused by spreading depression in the hippocampus similar to cortical spreading depression in migraine with aura has been developed in the 1980ies [3], until today no clear evidence could be found. To the contrary, although an abundance of studies—most of these single case reports—investigated perfusion abnormalities in the hippocampus in TGA by use of positron emission tomography (PET) or single-photon emission computed tomography (SPECT), their results are inconsistent and do not give a clear picture (see Table 1).

**Table 1:** Overview of studies examining perfusion alterations in TGA in the acute phase.

Article	Year	n	Method	Perfusion abnormalities
***SPECT***				
Trillet et al. [24]	1987	2	133Xe-SPECT	↓ global, more pronounced in the right temporal lobe (n = 1), or in the left frontal and temporal lobe
Stillhard et al. [23]	1990	1	99m-Tc-HM-PAO-SPECT	↓ temporal lobe bilaterally
Goldenberg et al. [49]	1991	1	99m-Tc-HM-PAO-SPECT	↓ global, marked in the thalamus bilaterally
Evans et al. [26]	1993	1	99m-Tc-HM-PAO-SPECT	↓ postero-medial temporal lobes bilaterally
Lin et al. [25]	1993	1	99m-Tc-HM-PAO-SPECT	↓ occipital lobe bilaterally, left medial temporal lobe, left thalamus
Kazui et al. [27]	1995	3	99m-Tc-HM-PAO-SPECT	↓ mesial temporal lobes bilaterally
Jung et al. [28]	1996	1	99m Tc-ECD-SPECT	↑ right medial temporal lobe
Sakashita et al. [75]	1997	2	99m-Tc-HM-PAO-SPECT	↑ occipital lobe, cerebellum
Schmidtke et al.[50]	1998	6	99m-Tc-HM-PAO-SPECT	↓ temporal (n = 4), as well as frontal (n = 3), parietal, and occipital lobes (n = 4), thalamus, and lentiform nucleus (n = 2) ↑ thalamus, and lentiform nucleus bilaterally (n = 1)
Jovin et al. [29]	2000	1	99m-Te-bicisate- SPECT	↓ mesial temporal lobes bilaterally
Warren et al. [30]	2000	1	99m-Tc-HM-PAO-SPECT	↓ right basal ganglia, left anterior temporal lobe
Nardone et al. [31]	2003	13	99m-Tc-HM-PAO-SPECT	↓ thalamus, corpus striatum (n = 13), unilateral or bilateral temporal lobe (n = 4)
Bucuk et al. [32]	2004	2	SPECT	↓ left frontal lobe and right medial temporal lobe (n = 1), medial temporal lobes bilaterally (n = 1)
Lampl et al. [33]	2004	16	99m-Tc-HM- PAO-SPECT	↓ temporal lobe uni- oder bilaterally (n = 11), thalamus (n = 1), basal ganglia (n = 1), occipital lobe (n = 1)
Takeuchi et al. [51]	2004	8	99m Tc-ECD- SPECT	↓ thalamus, and angular region
Tateno et al. [52]	2008	1	99m Tc-ECD- SPECT	↓ right thalamus
Yamane et al. [22]	2008	1	123-IMP- SPECT	↓ global (posteromedial temporal lobes, hippocampus, basal ganglia, and thalamus bilaterally)
Yang et al. [34]	2009	5	99m Tc-ECD- SPECT	↓ temporal lobes uni- or bilaterally, cerebellar vermis ↑ right corpus callosum, left frontal lobe
***PET***				
Baron et al. [53]	1994	1	^15^O-PET	↓ right lateral frontal lobe, thalamus, and lentiform nucleus
Eustache et al. [48]	1997	1	^15^O-PET	↓ left occipital lobe, thalamus, and lentiform nucleus
Guillery et al. [21]	2002	2	^15^O-PET	↓ right amygdala and left hippocampus (n = 1), left amygdale and hippocampus (n = 1)
***DSC-PWI***				
Budson et al. [4]	1999	1	DSC perfusion- weighted MRI	unremarkable
Greer et al. [5]	2001	1	DSC perfusion- weighted MRI	unremarkable
Toledo et al. [6]	2008	7	DSC perfusion- weighted MRI	unremarkable

Legend: n = number of patients.

PET and SPECT are used as reference methods for perfusion imaging but are not available in daily clinical routine. Furthermore, the application of PET and SPECT in clinical studies may be hampered by the fact that TGA patients are not able to give informed consent in the acute phase. On the other hand, multimodal MRI is the preferred imaging procedure in TGA, widely available, easily to perform even in the acute phase, and yields a surrogate of cerebral blood flow (CBF) and volume (CBV). Nevertheless, there are only a few reports on the application of DSC perfusion-weighted MRI (PWI) in TGA and all of these reported negative results [4–6]. However, meanwhile a substantial number of studies have been published addressing the detection of subtle perfusion abnormalities by use of PWI in different neurological disorders like migraine with aura [7,8], or crossed cerebellar diaschisis [9,10], an epiphenomenon in acute ischemic stroke.

In the present study, we sought to explore PWI findings in TGA patients in the acute phase (1) by visual inspection as well as (2) by a semi automatic imaging analysis in comparison to healthy control subjects.

## Material and Methods

### Patients and control subjects

The study was approved by the local institutional review board (Medizinische Ethikkommission II der Medizinischen Fakultät Mannheim). Patient consent was not required by our IRB for this de-identified database (Perfusion-weighted imaging in Transient Global Amnesia—PIT) due to the retrospective nature of the study and the lack of patient interaction. From a prospectively maintained MRI report database (Syngo Data Manager—SDM), we identified 698 patients with suspected acute ischemic stroke who underwent a standard stroke MRI protocol including PWI (2005–2013). For the present study we identified all those patients in whom clinical workup including multimodal MRI finally led to the diagnosis of transient global amnesia according to the established criteria of Hodges and Warlow for a TGA [11]. The demographic details and clinical presentation were abstracted from the case records. Patients who underwent a standard stroke MRI protocol including PWI to rule out an intracranial pathology (acute ischemic stroke, intracranial hemorrhage, or tumor) and unremarkable MRI findings served as control group. For each TGA patient, we selected three age and sex matched control subjects.

### MRI Studies

Magnetic resonance imaging was performed on a 1.5-T MR system (in 1 patient: Magnetom Sonata, in the remaining patients and control subjects: Magnetom Avanto, Siemens Medical Systems, Erlangen, Germany). The median time between onset of TGA and MRI was 4 hours (minimum 4 hours, maximum 9 hours). A standardized protocol was used in all patients including (1) transverse, coronal and sagittal localizing sequences followed by transverse oblique contiguous images aligned with the inferior borders of the corpus callosum (applied on sequences 2 to 5); (2) T1-weighted images (field of view 230x230 mm, acquisition matrix 512x512, number of slices 24, slice thickness 5 mm, TR 540/500 ms, TE 11/8.4 ms for Magnetom Sonata/Avanto); (3) T2-weighted images; (4) diffusion-weighted images (DWI); (5) fluid attenuated inversion recovery (FLAIR) images; (6) PWI following the first pass of contrast bolus through the brain; (7) T2*-weighted images, and (8) a 3D time-of-flight MR angiography (MRA). Perfusion-weighted imaging was acquired using a gradient-echo echo planar imaging sequence (field of view 230x230 mm, acquisition matrix 128x128, number of slices 12/19, slice thickness 6/5 mm, TR 1500/1430 ms, TE 46/30 ms, duration 1:30 minutes for Magnetom Sonata/Avanto). The contrast agent gadoteric acid (Dotarem, Guerbet, Aulnay-sous-Bois, France) was bolus injected by a power injector (Spectris MR injection system, Medrad, Volkach, Germany) with a dose of 0.1 mmol/kg of body weight at a rate of 4 ml/sec.

### Postprocessing

#### Perfusion maps

The postprocessing of the perfusion-weighted raw images was performed by a specific software, Signal Processing In NMR (SPIN, The MRI Institute for Biomedial Research, Detroit, USA, www.mrimaging.com) [12]. Deconvolution with singular value decomposition (SVD) was used to create quantitative maps of CBF, and CBV. The position of the arterial input function (AIF) was automatically determined by using the maximum concentration (Cmax), TTP and first moment MTT (fMTT). The concentration-time curve for arteries has short fMTT, short TTP and high Cmax. Twenty voxels, which best fitted these properties were selected. Then the concentration-time curves of these voxels were averaged, smoothed and truncated to avoid the second pass of the tracer. For the quantification of CBF and CBV, normal white matter served as an internal standard and was preset to 22 ml/100 g/min and 2.7 ml/100 g respectively [13–15]. Relative CBF/CBV values were calculated as ratios of mean CBF/CBV values in different volumes of interest (VOI) and mean CBF/CBV values in reference regions in the normal white matter.

#### Segmentation of subcortical structures

**Fig 1:**
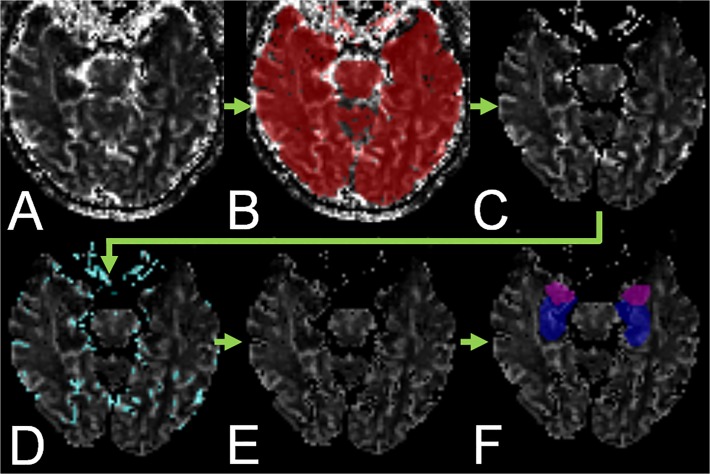
Schematic illustration of perfusion MRI postprocessing and analysis. A. CBF map. B. CBF map with superimposed parenchyma mask (red). C. Processed CBF map after application of the parenchyma mask. D. Processed CBF map with superimposed vessel mask (turquoise). E. Processed CBF map after application of the vessel mask. F. Processed CBF map with superimposed hippocampus VOI (blue) and amygdala VOI (violet).

Segmentation of subcortical structures (amygdala, hippocampus, thalamus, putamen, globus pallidus, and caudate nucleus) was performed by use of FIRST, a model-based segmentation tool in the Oxford Centre for Functional Magnetic Resonance Imaging of the Brain (FMRIB) Software Library (FSL) (www.fmrib.ox.ac.uk/fsl) [16]. Afterwards, the resulting masks were used to create corresponding volumes of interest (VOI, see Fig. 1) in a semiautomatic image display program (MRIcron, www.mccauslandcenter.sc.edu/mricro/mricron) [17].

#### Parenchyma and Vessel Mask

A parenchyma binary mask was obtained from the T1-weighted images by use of FAST (FMIRB Software Library; www.fmrib.ox.ac.uk/fsl) [18]. Since the extracted parenchyma voxels also included voxels containing vessels, a vessel binary mask was created from the Cmax maps. The parenchyma mask was applied prior to obtaining the voxel histogram of this image. Concentrations greater than 3.0 times the median in the histogram defined voxels containing vessels. This threshold was empirically chosen such that all vessels were extracted without including parenchyma voxels.

#### MRI Analysis

Magnetic resonance images were analyzed by two neuroradiologists (A.F., M.A.) in consensus. First, PWI was analyzed visually, with the readers blinded to the clinical information and all other sequences. For the analysis of CBF and CBV values in the subcortical structures, the parenchyma mask and the vessel mask were applied to the CBF and CBV maps such that all voxels within the parenchyma mask but not in the vessel mask were included (see Fig. 1). Then, the created VOI for all segmented subcortical structures were placed consecutively on the coregistered CBF and CBV maps and the mean CBF and CBV values were noted.

An additional acute or chronic cerebral pathology or an intracranial vascular pathology was excluded on DWI, T1-, T2-, T2*-weighted images, FLAIR images, and MR angiography respectively. The degree of white matter lesions (WML) severity on MRI was rated on FLAIR images using a modified version of the visual scale of Fazekas [19], that scores deep and subcortical WML in three categories of mild, moderate, and severe WML.

#### Statistical Analysis

Statistical analysis was carried out using SPSS 17.0. Descriptive data was analyzed using either t-tests, the rank-sum test or chi^2^ based tests as appropriate. Group comparisons for possible differences in rCBF and rCBV between TGA patients and control subjects were performed using the Mann-Whitney-U-Test. Furthermore, group comparisons for possible side differences in rCBF and rCBV in TGA patients were performed using the Wilcoxon-Test. All statistics was performed with a 0.05 level of significance.

## Results

### Patient demographics

Of the 5 TGA patients, 2 were men, and 3 were women. The descriptive data is displayed in Table 2. Two patients had vascular risk factors: one patient had arterial hypertension, the other patient hyperlipidemia and ischemic heart disease. One patient also had a migraine without aura.

**Table 2:** Clinical characteristics of TGA patients.

Patient	Age (y)	Sex	Vascular risk factors	Other diseases	Time between onset and MRI (h)	DWI lesion
**1**	61	M	-	-	9	-
**2**	63	F	Hypertension	Migraine	4	Left hippocampus
**3**	66	F	Hyperlipidemia, ischemic heart disease	Vestibular neuropathy	4	-
**4**	69	F	-	Hysterectomy, breast cancer	7	-
**5**	73	M	-	-	4	-

Legend: y = years, h = hours, M = male, F = female

### MRI findings

**Fig 2:**
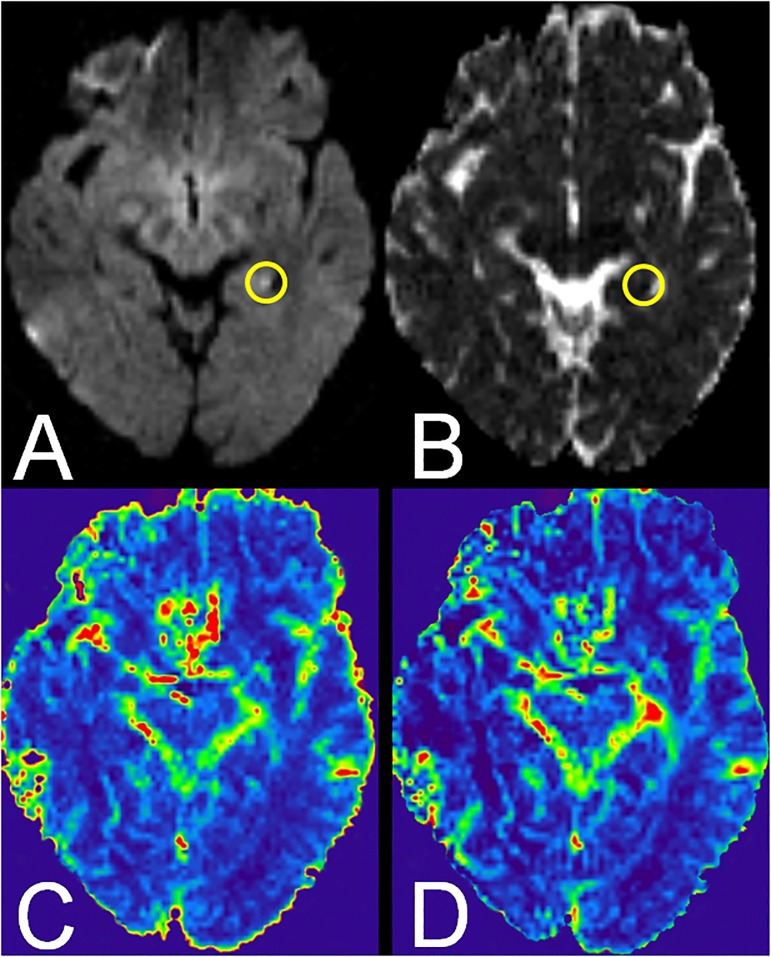
MRI findings in TGA patient 2. A. Focal DWI hyperintensity in the left hippocampus on axial DWI. B. Corresponding decrease of ADC on ADC map. C. CBF and D. CBV are unremarkable.

On DWI, in only one TGA patient (patient 2) an acute lesion was observed in the lateral aspect of the left hippocampus (see Fig. 2). In the other four patients DWI was unremarkable in the acute phase. Regarding the extent of chronic WML, three patients and ten control subjects had no WML (Fazekas grade 0) while two patients and five control subjects had mild WML (Fazekas grade 1).

On PWI, no relevant perfusion alterations were found by visual inspection in TGA patients. Group comparisons for possible differences between TGA patients and control subjects showed significant lower rCBF values bilaterally in the hippocampus, in the left thalamus and globus pallidus as well as bilaterally in the putamen and the left caudate nucleus. In the right thalamus we observed only a trend towards a lower rCBF (see Table 3). In contrast, no significant differences in rCBF values were found in the amygdala bilaterally and the right globus pallidus.

**Table 3:** Group comparisons of rCBF data in TGA patients and control subjects (median (IQR)).

Regions	TGA patients	Control subjects	P
**R Amygdala**	31.66 (29.10–42.39)	39.40 (35.80–44.23)	0.09
**L Amygdala**	35.12 (28.78–39.40)	38.63 (35.09–41.72)	0.15
**R Hippocampus**	34.61 (32.02–43.74)	46.89 (42.10–52.96)	**0.03**
**L Hippocampus**	38.40 (31.99–43.54)	43.84 (41.09–47.97)	**0.04**
**R Thalamus**	39.60 (35.12–49.35)	49.42 (43.31–55.11)	0.05
**L Thalamus**	41.20 (36.74–48.93)	51.34 (45.79–56.04)	**0.04**
**R Putamen**	37.14 (34.02–45.21)	50.24 (44.56–58.10)	**0.01**
**L Putamen**	35.55 (31.07–45.94)	49.87 (42.87–56.28)	**0.02**
**R Globus pallidus**	33.89 (31.46–36.70)	36.14 (34.40–39.38)	0.15
**L Globus pallidus**	34.67 (31.74–35.53)	38.62 (34.90–44.16)	**0.04**
**R Caudate nucleus**	35.92 (34.13–47.40)	47.66 (46.44–55.11)	**0.03**
**L Caudate nucleus**	34.57 (32.97–45.63)	49.20 (43.96–55.96)	**0.01**

Legend: R = right, L = left

Correspondingly, significant lower rCBV values were observed bilaterally in the hippocampus and the putamen as well as in the left caudate nucleus (see Table 4) while in all other subcortical structures no significant differences in rCBV values were found.

**Table 4:** Group comparisons of rCBV data in TGA patients and control subjects (median (IQR)).

Regions	TGA patients	Control subjects	P
**R Amygdala**	3.82 (3.26–5.34)	4.78 (4.34–5.56)	0.15
**L Amygdala**	4.54 (3.34–4.72)	4.88 (4.24–5.28)	0.60
**R Hippocampus**	4.85 (4.23–6.13)	6.61 (5.80–6.87)	**0.04**
**L Hippocampus**	4.97 (4.01–5.89)	6.12 (5.49–6.57)	**0.04**
**R Thalamus**	5.88 (4.57–6.28)	6.26 (5.64–7.04)	0.13
**L Thalamus**	5.90 (4.89–6.43)	6.21 (5.80–6.90)	0.22
**R Putamen**	4.88 (4.33–5.39)	6.18 (5.32–6.96)	**0.01**
**L Putamen**	4.89 (3.72–5.43)	5.91 (5.42–6.58)	**0.01**
**R Globus pallidus**	4.02 (3.67–4.28)	4.44 (3.75–4.92)	0.15
**L Globus pallidus**	4.15 (3.46–4.49)	4.40 (4.01–5.02)	0.24
**R Caudate nucleus**	5.54 (4.60–6.31)	6.11 (5.89–7.10)	0.09
**L Caudate nucleus**	5.21 (4.45–5.48)	6.26 (6.06–7.01)	**0.003**

Legend: R = right. L = left

Group comparisons for possible side differences in rCBF and rCBV values in TGA patients revealed only a significant lower rCBV value in the left caudate nucleus (p = 0.042) while in all other subcortical brain regions no significant differences could be detected.

## Discussion

The underlying pathophysiology of TGA is still unexplained and the relevance of perfusion abnormalities as demonstrated in several case reports and smaller case series remains unclear. In the present study, we used a semi automatic image analysis approach to detect subtle PWI changes in several subcortical brain structures in TGA patients in comparison to normal control subjects. Interestingly, we observed significant lower rCBF and rCBV values in the hippocampus bilaterally in TGA patients in comparison to control subjects. With regard to the clinical syndrome, the hippocampus is the anatomical structure most likely affected by a decreased cerebral blood flow in TGA. Furthermore, the only detectable MRI findings in TGA patients are small, circumscribed lesions in the lateral hippocampus usually occurring with a latency period of 24 to 72 hours [20]. Nevertheless, unilateral or bilateral hypoperfusion in the hippocampus was only demonstrated in a subset of studies using PET or SPECT [21,22] while a hypoperfusion in the temporal lobe in general was much more common [23–34].

Additionally, we found lower rCBF values in the left thalamus, the left globus pallidus and bilaterally in the putamen and the caudate nucleus in TGA patients in comparison to control subjects. Comparable results have been published in case reports and case series using PET and SPECT (see Table 1). On first glance, this finding may appear counterintuitive since the clinical presentation of TGA indicates primarily involvement of the hippocampal formation. However, the thalamus has been related to memory deficits for quite some time [35,36] and there has been growing evidence that even the putamen and the caudate nucleus are involved in memory processes such as maintaining and manipulating information [37,38]. In acute ischemic stroke limited to the thalamus impairment of memory is a common clinical symptom in particular in the territory of the tuberothalamic artery, the paramedian artery, and the posterior choroidal arteries [39]. Acute ischemic stroke in the putamen may cause short term memory deficits besides other symptoms like hemiparesis, movement disorders, aphasia, and hemineglect [40]. Similarly, acute ischemic stroke in the caudate nucleus may be accompanied by various memory deficits [41,42]. Even case reports have been published describing cases of TGA patients with an ischemic lesion or intracerebral hemorrhage in the left putamen [43], right caudate nucleus [44], left lentiform nucleus [45] or thalamus [46,47]. Regarding perfusion imaging with PET or SPECT, several studies reported unilateral or bilateral hypoperfusion in the thalamus [22,25,31,33,48–52] as well as in the putamen and caudate nucleus, in most of these studies referred to as lentiform nucleus [48,50,53] or corpus striatum[31].

On visual inspection, we found no evidence of perfusion changes in patients with TGA which is in line with earlier studies reporting that perfusion abnormalities are rarely found on PWI in TGA patients in the acute or subacute phase [4–6]. Furthermore, in the study of Toledo and colleagues the observed areas of hypoperfusion could always be attributed to coincident vascular pathologies like anterior, middle or posterior cerebral artery stenosis [6]. Thus, qualitative assessment of CBF and CBV maps by visual inspection may be regarded as insufficient to detect perfusion alterations in TGA patients.

The nature of the observed perfusion alterations is still unclear. In particular the question whether a reduction of cerebral perfusion causes the memory disturbance or represents only an epiphenomenon of TGA remains unanswered. In 1986, Olesen and Jørgensen suggested spreading depression in the hippocampus as a possible pathomechanism in TGA [3]. The hippocampus is highly susceptible to spreading depression and spreading depression has been elicited in animal experiments [54,55] as well as in the human hippocampus in vitro [56] and in vivo [57]. Since then, shared pathophysiological mechanisms of migraine with aura and TGA have repeatedly been suggested. The pathophysiological basis of migraine aura is the cortical spreading depression, a state of spreading electrical discharge and depolarization of neurons and glia followed by complete recovery of cortical electrical activity. The phenomenon is usually accompanied by a hyperperfusion for about 1–2 minutes followed by a mild hypoperfusion for about 1–2 hours [58,59]. In patients with migraine with aura perfusion abnormalities can be detected reliably by visual inspection of PWI in more than 50% of patients [7,8]. And even hypoperfusion following spreading depression in the thalamus and the globus pallidus has been observed[60]. However, comparable studies on perfusion alterations in the amygdala, hippocampus, basal ganglia, or thalamus in humans have not been published yet. In this light the observed perfusion alterations in TGA possibly might represent the subsequent mild hypoperfusion after cortical spreading depression.

Another explanation might be a secondary hypoperfusion following a neural depression in various subcortical brain regions caused by transitorily impaired connections to the hippocampus independent of the primary pathology comparable to the concept of diaschisis [61]. Although in most studies PET has been used to demonstrate a hypometabolism or hypoperfusion in brain areas distant from the actual lesion like the ipsilateral cerebral cortex [62,63] or the contralateral cerebellum [64–68], diaschisis has also been detected by PWI in several cerebral lesions, such as acute ischemic stroke [9,10]. Only recently, Peer and colleagues very elegantly demonstrated a reversible disturbance of functional connectivity in TGA patients by use of resting state functional MRI [69]. The observed connectivity changes were bilaterally, more pronounced in the acute phase of TGA, and decreased stepwise within the postacute phase.

However, in both suggested pathomechanisms, cortical spreading depression as well as diaschisis, perfusion alterations can be regarded as concomitant phenomena rather than the underlying cause of TGA.

The present study has some limitations. First, this is a retrospective clinical study of small sample size. However, to our knowledge this is the only study investigating perfusion alterations on PWI in a series of TGA patients in the acute phase in detail. Thus, the presented study results may be regarded as preliminary and should encourage future studies using PWI in TGA patients. Second, perfusion changes in TGA might develop in different stages affecting various brain regions. As a consequence perfusion changes could be underestimated in group comparisons when patients were not examined at same stages of development. In future studies perfusion alterations should be assessed in different patient groups categorized by the time between onset of TGA and MRI. Third, automatic segmentation is never perfect and may lead to minor inaccuracies in defining the borders of subcortical brain structures [70,71]. On the other hand the major advantage of an automatic segmentation is the possibility of an investigator independent analysis. Fourth, since CBF and CBV vary between different subjects dependent on age, gender, and other factors [72,73], we cannot completely exclude an influence of individual factors on the determined relative CBF and CBV values in the present study. Nevertheless, interindividual variations in CBF and CBV should be reduced by matching each TGA patient with three control subjects of same sex and similar age. Further factors in turn such as blood pressure seem negligible since the cerebral blood flow remains relatively constant in normotensive subjects even at a higher age due to cerebral autoregulation [74]. Fifth the hospital-based retrospective study design might cause several types of bias and statistical errors such as selection bias, sample bias, or image-based selection bias. Finally, follow-up PWI was not available in the included TGA patients and consequently, no information about the evolution of perfusion abnormalities could be obtained.

In conclusion, visual inspection of PWI is not appropriate for the assessment of associated perfusion changes in TGA in the acute phase but still might be useful to rule out incidental intracranial vascular pathologies in daily routine care. Semi automatic image analysis in comparison to healthy control subjects might be useful to detect subtle perfusion changes on PWI in TGA patients. However, this should be confirmed in larger data sets and serial MRI examinations including PWI. With regard to the benign nature of TGA, the fact that neuroimaging is not generally recommended for the diagnosis, and rare but severe complications after contrast agent application (nephrogenic systemic fibrosis) alternative MR perfusion measurement techniques, such as arterial spin labeling should be evaluated in future studies. This technique allows an absolute quantification of CBF and intravenous administration of a contrast agent is not necessary.
